# An economical strategy for early medical education in ultrasound

**DOI:** 10.1186/s12909-018-1275-2

**Published:** 2018-07-18

**Authors:** Alexandra Mullen, Brendan Kim, Jose Puglisi, Nena Lundgreen Mason

**Affiliations:** 1 Department of Basic Sciences, California Northstate University College of Medicine, 9700 West Taron Drive, Elk Grove, California, 95757 USA; 2 Department of Biomedical Sciences, Rock Vista University College of Osteopathic Medicine, 255 East Center Street, Ivins, UT 84738 USA

**Keywords:** Medical education, Ultrasound education, Economical ultrasound training, Undergraduate medical education

## Abstract

**Background:**

A movement to include ultrasound training in undergraduate medical education is slowly taking place. However, many educational institutions are hesitant to include formal ultrasound training as a part of their curricula due to curricular time constraints, high cost of ultrasound equipment, and a lack of sufficient faculty skilled with ultrasound. We suggest that an economical ultrasound training strategy is needed to resolve these obstacles and enable hesitant medical programs to include ultrasound training.

**Methods:**

Twenty-eight first year medical students volunteered to attend extra-curricular ultrasound training sessions covering topics related to 11 commonly used sonographical imaging categories. Study assessments included subjective pre/post-training skill evaluation surveys, and objective numerical scores awarded by the session instructor during real-time evaluation of each participant’s performance in obtaining each target ultrasound view.

**Results:**

A Wilcoxon matched-pairs signed rank test was performed to evaluate the difference between pre-training and post-training survey questions. *P* values < 0.05 were considered significant. Moreover, following analysis the *p* value for all test was found to be < 0.0001. Of the 308 total ultrasound-related tasks attempted collectively by all 28 participants, only 7 (2.3%) tasks were deemed unsuccessful by an instructor.

**Conclusions:**

The training program presented in this study requires one faculty member, a single ultrasound machine, and time to conduct six 30-min training sessions with small groups of students over 4 weeks. Many medical schools are concerned that they don’t have adequate time or resources to include ultrasound training in their curricula. Our intention is to negate these concerns by providing a simple and practical training method that is both temporally and fiscally economical.

## Background

The field of clinical ultrasonography has expanded a great deal in the past decade. Ultrasound technology is being utilized across a growing number medical and surgical specialties as an adjunct for establishing bedside diagnoses, and for image guided procedures [1, 2]. The ultrasound machine is already a valuable adjunct to auscultation, and has in fact largely replaced the stethoscope in the fields of cardiology [3, 4]**,** Obstetrics [5]**,** and Gastroenterology [6–10]**.** The already widespread usage of ultrasonography in cardiology, obstetrics, and radiology is transcending into fields such as internal medicine, emergency medicine, surgery, anesthesiology, critical care, and numerous other specialties [11]. The advent of portable and handheld ultrasound devices has paved the way for the burgeoning field of focused ultrasonography, where physicians can simultaneously perform and interpret imaging studies at the bedside [11]. This growing use of point-of-care ultrasonography across a variety of clinical disciplines has highlighted the need to ensure a high level of competence in ultrasonography among physicians across many specialties [12].

There have been movements within educational institutions and accrediting bodies to begin implementing ultrasound competency requirements for medical students and residents. For example, in 2013 the Accreditation Council for Graduate Medical Education (ACGME) approved two ultrasound-related program requirements for graduate medical education in emergency medicine. Because of these new competencies and an increased use and availability of ultrasound technology in many medical disciplines, there is a great need to train a new generation of physicians that are capable and confident with ultrasound. A few medical schools have now integrated ultrasound into all four years of their medical training [13], enabling their students to be leaders and teachers of ultrasound in both their clerkships and residencies [14–16]. These institutions have reported that many kinds of formal ultrasound training significantly increase competency in ultrasound fundamentals for both medical students [13–15] and residents [17]. There is evidence that with as little as eight weeks of training, it is possible to teach a medical trainee the fundamentals of ultrasonography. Following eight weeks of training medical students were able to identify commonly occurring pathologies in outpatient clinics which lead to reduced costs, patient wait times, pressure on their radiology departments, and improvement in their individual diagnostic capability [18].

Mainstream inclusion of ultrasonography as a core component of undergraduate medical education has been slow due to curricular time constraints, high cost of ultrasound equipment, and a lack of sufficient faculty skilled with ultrasound [13]. The inundated state of medical school curricula has been a central topic of discussion within the academic community for the past hundred years [19]. Matters have exponentially worsened in this regard as medical science has advanced and generated more for students to learn [20, 21]. This concept makes the thought of finding a place for even eight weeks of ultrasound training a daunting task for medical schools, despite the many valuable applications of this technology. Additionally, a lack of proper faculty training, coupled with high costs of equipment and instruction, deter programs from making ultrasound training mainstream in medical schools [11]. A few of the programs teaching ultrasound to medical students have arranged instruction to follow and augment the pertinent lessons in anatomy, physiology, and physical exam [1]. These early courses in ultrasound have been proven to increase students’ competence and confidence in using ultrasound [11]. However, specific standards for evidence-based methods in teaching ultrasound at the undergraduate level have yet to be defined [11].

## Methods

### Participants and study design

This study was conducted at an accredited United States medical school. Prior to beginning this study, all of its components were reviewed and approved by the appropriate institutional review board. Twenty-eight first-year medical students from the student body volunteered and consented both verbally and in writing to attend a series of ultrasound training sessions and participate in this study. Study participants were allowed to sign up in small groups for training sessions at their convenience outside of scheduled class/lab times over a period of four weeks. All ultrasound training sessions were led by a single faculty member skilled in point-of-care ultrasound for an average of 8 h of faculty teaching time per week. Faculty teaching time varied from 6 to 10 h per week, with an average of 8 h per week due to the flexible extra-curricular scheduling methodology used.

The study was comprised of two main components. The first required participation in six hands-on ultrasound training sessions of 30 min each (see Table 1) that covered 11 commonly used sonographical views; the second component required participants to complete a survey prior to the initial training session and following the final training session. Both the pre and post-training surveys assessed each participant’s subjective confidence level in their ability to perform various basic ultrasound related tasks (see Table 2) using a Likert scale (1 = strongly disagree to 5 = strongly agree). Survey questions were also included to assess time participants had previously spent operating an ultrasound machine to determine if they had received additional training prior to the start of, or during this study. Additionally, participants were given an opportunity report both their perceptions regarding their experience in our ultrasound sessions, and the effectiveness of these sessions, following completion of the training program on the post-training survey (see Table 2, questions 21–24). The curriculum contained no additional learning time, assignments, or readings other than the hands-on training sessions themselves. No textbook was used.

**Table 1:** Ultrasound Training Program Curriculum

Session	Tasks and Topics
1	Basic Knobology (Machine Operation) Power on the machine Select appropriate scan setting Adjust gain and depth Demonstrate appropriate indicator orientation Image capture General strengths and weakness of various probes Abdominal aorta, Inferior vena cava, and carotid artery Transverse and longitudinal plane transition
2	Right and left kidney Morison’s pouch and splenorenal space Transverse and longitudinal renal plane transition
3	Subxiphoid cardiac window Apical four-chamber cardiac window
4	Parasternal long axis of the heart Parasternal short axis and ejection fraction evaluation
5	Lung parenchyma evaluation (pulmonary edema) Pleural line evaluation (pneumothorax and hemothorax) Recognize the difference between A & B lines Costodiaphragmatic recess fluid evaluation
6	Gall Bladder Urinary bladder & volume measurement

Six ultrasound training sessions and the corresponding tasks and topics covered in each session

**Table 2:** Subjective Evaluation Questions

1. I can successfully turn on an ultrasound machine and reach the exam screen without help	
2. I can optimize the gain of an ultrasound image to maximize clarity while maintaining correct image contrast	
3. I can adjust the depth of an ultrasound image to optimize image clarity for both deep and superficial structures	
4. I feel confident that I can use an ultrasound machine to measure the diameter of a structure	
5. I feel confident that I can obtain clear images of the heart using the parasternal long axis view	
6. I feel confident I can obtain clear images of the heart using the parasternal short axis View	
7. I feel confident that I can obtain clear images of the heart using the apical 4-chamber view	
8. I feel confident that when conditions are optimal, I can obtain clear images of the heart using the subxiphoid view	
9. I know which direction the probe indicator should be placed relative to the patient in all 4 cardiac views	
10. I know which direction the probe indicator should be placed in sonography of the lungs	
11. I feel confident that I can obtain clear images of the lung	
12. I feel confident that I can identify the “ants on log” a sign in lung tissue	
13. I feel confident that I can identify the both A lines and B lines on lung ultrasound	
14. I feel confident that I can obtain clear images of the abdominal aorta in both transverse and longitudinal orientations	
15. I feel confident that I can obtain clear images of the inferior vena cava in the abdomen	
16. I feel confident that I can obtain clear images of the right and left kidney in both longitudinal and transverse views	
17. I feel confident that I can locate the splenorenal space and Morison’s pouch on renal ultrasound	
18. I feel confident that I can obtain clear images of the gall bladder	
19. I feel confident that I can obtain clear images of the bladder	
20. I know which direction the probe indicator should be placed in sonography of the abdomen	
(Note: questions 21–24 were included on the post-training survey only) 21. I feel that these ultrasound training sessions were enjoyable 22. I feel these ultrasound training sessions effectively taught me overall basic tactile ultrasound skill 23. I feel that ultrasound training sessions like those I just completed are beneficial to first-year medical students 24. Please include any other comments you may have about your experiences during these ultrasound training sessions in the space provided	

The listed questions were presented to participants before and after ultrasound training. A Likert scale was used in the evaluation of each participant’s own subjective level of confidence in performing specific tasks in basic ultrasound skill. The following scale was used: 1 = strongly disagree, 2 = disagree, 3 = somewhat agree, 4 = agree, 5 strongly agree

### Ultrasound training sessions

Participants volunteered to serve as scanning subjects within their groups (of 2–4) and were required to sign a consent form to do so. To protect participant privacy no ultrasound images of participant anatomy were printed or saved. Each ultrasound training session covered a few of the 11 commonly used sonographic views listed below. See Table 1 to see which sonographic views were covered in each specific training session. Each of the six 30-min training sessions began with a 10-min verbal explanation of pertinent anatomy and probe handling technique from the session instructor. Next, the participants were given approximately 20 min for all participants in the group to take turns attempting to acquire diagnostic quality images of all the sonographic views covered in that particular training session. Participants were given verbal instruction as needed to help them acquire the images correctly, but participants were largely allowed to learn tactile skill and probe handling by trial and error.

1. Parasternal long axis view of the heart
2. Parasternal short axis view of the heart
3. Apical four chamber view of the heart
4. Subxiphoid view of the heart
5. Jugular vein and carotid artery
6. Lung parenchyma and pleural membranes
7. Right kidney and Morison’s pouch
8. Left kidney and splenorenal space
9. Bladder and volume measurement
10. Gall bladder
11. Inferior vena cava and abdominal aorta

During each session, every effort was made to minimize time spent on instructor demonstration. This provided participants maximal time to acquire each target view themselves. To objectively ensure that each view/image acquired was of diagnostic quality, each individual participant was carefully evaluated by the instructor, in real time, during every scan. Participants were asked to hold each target view long enough for evaluation by the session instructor. A numerical score was then awarded, indicating the quality of images obtained. Protocol for instructor evaluation was modeled after the method described by Evans and Evans in 2015 [18]. Possible scores included; 0: failure to visualize target view/organ, 1: organ/view visualized but image quality is poor, and 2: clear diagnostic quality demonstration of view/organ.

### Equipment and cost

All ultrasound scans were performed using a single GE LOGIQ e R7 (GE Healthcare, Chicago, IL, USA) ultrasound machine. The ultrasound machine was equipped with standard phased array (3 MHz), linear (12 MHz), and curvilinear (1-5 MHz) probes (also by GE).The price of this model of ultrasound machine and probes vary by location and by intended use (hospital setting vs. educational setting). The machine and probes used in this study were purchased by the university for educational purposes from GE for 37,000$. Additional study equipment included approximately 3 quarts of Aquasonic transmission gel (ParkerLabs) and towels, which totaled in cost to 50$. All training sessions were either held in a hospital simulation room or in a small classroom on campus. Because these campus facilities are used for medical skills sessions, ultrasound sessions were only scheduled when the rooms were unoccupied. The cost of these rooms was not included in the overall cost of running this project since they are not dedicated spaces for ultrasound training. There were no additional funding sources for this project.

### Statistical analysis

Prior to statistical analysis, participant names were disassociated from all data and replaced with randomly generated alphanumeric codes via a customized software using LabVIEW 2016 by National Instruments Inc. A Wilcoxon matched-pairs signed rank test was performed to evaluate the difference between pre-training and post-training survey questions using GraphPad Prism7 (Graph Pad Software Inc., La Jolla, CA, USA.). *P* values of < 0.05 were considered significant. Moreover, following analysis the *p* value for all tests was found to be < 0.0001 therefore, all reported Wilcoxon tests were considered significant. Tables 3, 4, 5 and 6 which can be found in the supplementary material, numerically illustrate the median, minimum, and maximum for questions 1-20 shown in Table 2.

**Table 3:** Descriptive statistics of pre and pos questions 1, 2, 3 and 4

	Q1		Q2		Q3		Q4	
	(Pre)	(Pos)	(Pre)	(Pos)	(Pre)	(Pos)	(Pre)	(Pos)
Min	1	3	1	3	1	3	1	3
Max	5	5	5	5	5	5	5	5
Median	1	5	1	5	1	5	1	5
*P*	< 0.0001		< 0.0001		< 0.0001		< 0.0001

**Table 4:** Descriptive statistics of pre and pos questions 5, 6, 7, 8 and 9

	Q5		Q6		Q7		Q8		Q9	
	(Pre)	(Pos)	(Pre)	(Pos)	(Pre)	(Pos)	(Pre)	(Pos)	(Pre)	(Pos)
Min	1	3	1	3	1	3	1	3	1	3
Max	3	5	3	5	3	5	5	5	5	5
Median	1	5	1	5	1	5	1	5	1	5
*P*	< 0.0001		< 0.0001		< 0.0001		< 0.0001		< 0.0001

**Table 5:** Descriptive statistics of pre and pos questions 10, 11, 12, 13,14, and 15

	Q10		Q11		Q12		Q13		Q14		Q15	
	(Pre)	(Pos)	(Pre)	(Pos)	(Pre)	(Pos)	(Pre)	(Pos)	(Pre)	(Pos)	(Pre)	(Pos)
Min	1	3	1	3	1	4	1	4	1	1	1	1
Max	5	5	2	5	3	5	2	5	4	5	4	5
Median	1	5	1	5	1	5	1	5	1	5	1	5
*P*	< 0.0001		< 0.0001		< 0.0001		< 0.0001		< 0.0001		< 0.0001

**Table 6:** Descriptive statistics of pre and pos questions 16, 17, 18, 19 and 20

	Q16		Q17		Q18		Q19		Q20	
	(Pre)	(Pos)	(Pre)	(Pos)	(Pre)	(Pos)	(Pre)	(Pos)	(Pre)	(Pos)
Min	1	3	1	3	1	4	1	4	1	1
Max	5	5	2	5	3	5	2	5	4	5
Median	1	5	1	5	1	5	1	5	1	5
*P*	< 0.0001		< 0.0001		< 0.0001		< 0.0001		< 0.0001	

## Results

### Subjective assessment: Participant survey data

**Fig. 1:**
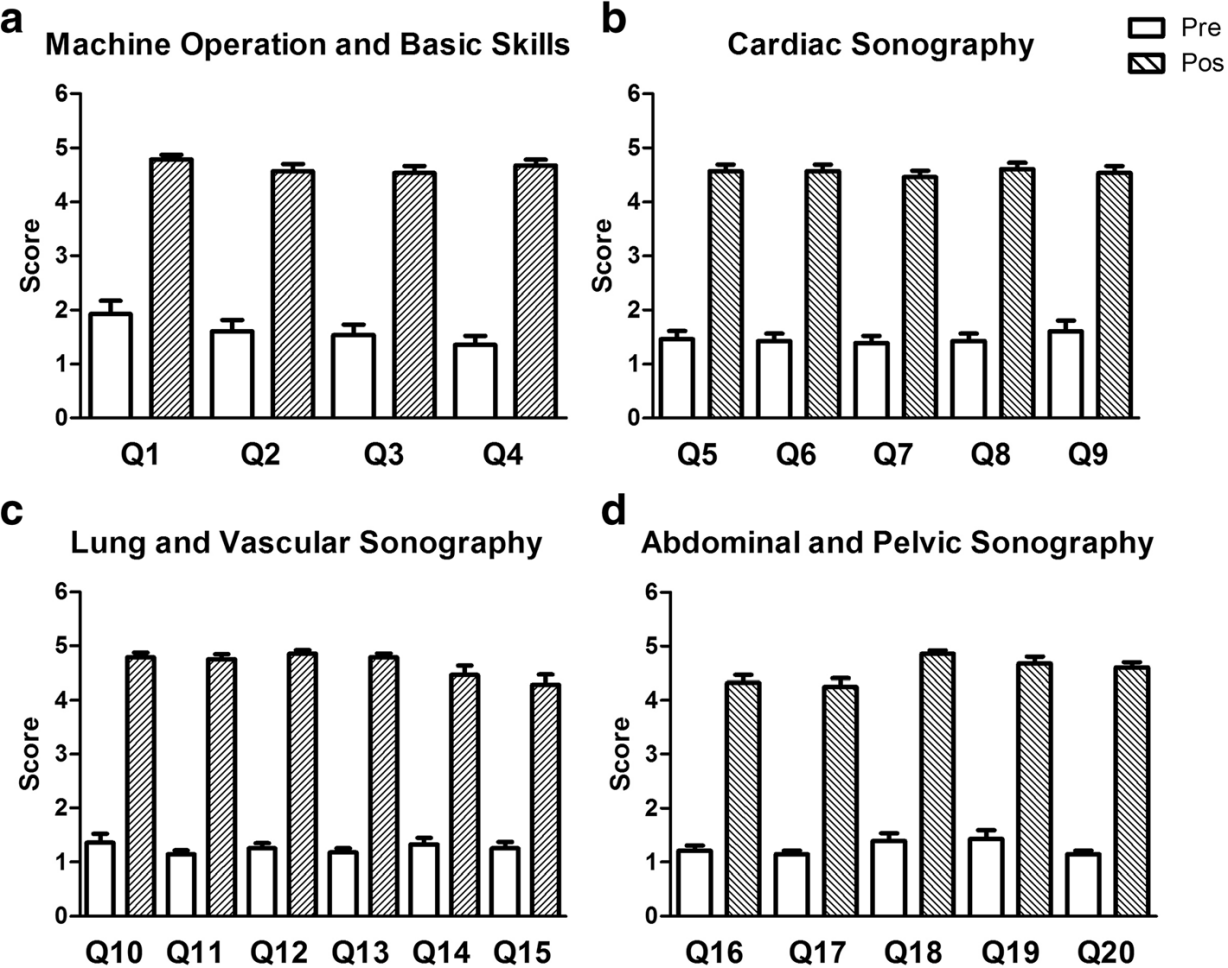
Subjective Reporting of Participant Confidence in Basic Ultrasound Skill. Four sets of bar graphs are displayed. The unshaded bars represent the average response among participants on pre-training survey questions, and the shaded bars represent the average response reported on post-training survey questions. Panel **a** shows the average responses to questions pertaining to machine operation and basic skills (See Table 2, questions 1–4). Panel **b** reports data from cardiac sonography questions (see Table 2, questions 5–9). Panel **c** contains data from questions pertaining to sonography of the lungs and vasculature (see Table 2, questions 10–15). Panel **d** reports data from questions regarding abdominal and pelvic sonography (see Table 2, questions 16–20)

The results of participants’ subjective responses to pre and post-session surveys are reported in Fig. 1. Each survey was comprised of 20 questions. Questions from the survey that evaluated specific types of ultrasound skill were grouped together during analysis and are displayed separately in each panel of Fig. 1. Panel A shows the average responses to questions pertaining to machine operation and basic skills (See Table 2, questions 1–4). Panel B reports data from cardiac sonography questions (see Table 2, questions 5–9). Panel C contains data from questions pertaining to sonography of the lungs and vasculature (see Table 2, questions 10–15). Panel D reports data from questions regarding abdominal and pelvic sonography (see Table 2, questions 16–20).

Panel A shows that the subjective confidence level of participants in basic machine operation and skill increased from an average of two (disagree) before training sessions to five (strongly agree) post-training. Panel B shows participant responses pertaining to cardiac sonography increased from 1.5 to five. Panels C and D show participant responses in all remaining categories increased from one to five following ultrasound training.

The unshaded bars represent participant responses to pre-training survey questions which asked them to evaluate their subjective level of confidence in their ability to perform a series of ultrasound-related tasks independently. Across all pre-session questions, participants reported that they disagreed or strongly disagreed that they were capable for performing the tasks covered during our training program. The shaded bars display data compiled from the post-session surveys. The shaded bars indicate that following training, participants largely strongly agreed that they could successfully complete all covered ultrasound-related tasks independently. There was approximately a 2.5-fold increase in participant confidence level following completion of our ultrasound training program.

An additional survey question on the pre-training survey evaluated the level of experience participants had prior to our study. Out of the 28 students that elected to participate in this study, 78.6% had no previous experience operating an ultrasound machine independently, 14.3% had 1–3 h of experience, and the remaining 7.1% had more than 10 h of experience. None of the participants received any outside training or had additional practice apart from the six training sessions we provided during the course of this study. We found no statistically significant difference in results between participants who had some prior training and completely untrained participants in our subjective or objective data. Additionally, data from questions 21–23 on the post-training survey showed that 96% of participants strongly agreed that our training sessions are particularly beneficial to medical students when delivered during the very first year of medical school. Additionally, 96% reported that they strongly agreed that participation in our ultrasound training program was very enjoyable. 86% of participants strongly agreed that the training sessions effectively taught fundamental ultrasound skills. The remaining 14% of participants agreed with this statement.

Participants were also given an opportunity to submit any additional comments regarding their experience in our ultrasound sessions following completion of the training program on the post-training survey (see Table 2, question 24). Participant comments contained overwhelmingly positive feedback. Participants reported statements like “Learning these (ultrasound) skills as a first year medical student helped me hone my technique and feel prepared for clinical rotations” and “Learning these basic ultrasound techniques has really helped me integrate classroom material with ultrasound images”. One participant also commented, “This should definitely be taught to all first-years because it adds another dimension of depth to anatomy and pathology in that we get an idea of what normal and abnormal looks like in a real person.”

### Objective assessment: Instructor scoring

Twenty-four of the 28 participants were able to score a 2 (image of satisfactory diagnostic quality) across all tasks they were asked to perform during our training program (see Table 1). The remaining four participants either scored a 0 (completely failed to obtain the target image) or a 1 (organ/view visualized, but image quality is poor) when attempting 1–2 of the 11 total tasks they were each asked to perform during our training program. However, those same four participants were able to score 2 on the remaining 9–10 ultrasound tasks. Of the 308 total tasks attempted collectively by all 28 participants, only 7 (2.3%) tasks were unsuccessful (received scores below 2).

## Discussion

### General conclusions

In this study, we present an evaluation of a novel and economical ultrasound training program. This program was designed to negate many of the barriers that prevent hesitant schools from integrating ultrasound training into their medical education programs. During this study, we were able to train 28 students to acquire high quality images of 11 commonly used sonographic views in six 30-min sessions over four weeks. Only a single faculty member and one ultrasound machine were needed to administer this training program. This ultrasound training strategy could easily be administered as a short elective course and yield up to 30 (there were 30 students who received training, however, only 28 fully participated in our study) medical students per academic quarter who are confident and objectively competent with the basics of ultrasound use across multiple clinical disciplines. The series of short training sessions described in this study will provide students with the fundamental skills and confidence needed to easily transition into more rigorous diagnostic scanning that is increasingly being required in modern clerkships and residencies, while also being conservative with faculty time and university resources.

### Assessments

Objective and subjective data presented in this study indicate that medical students are very capable of acquiring diagnostic quality images across many areas of the body with minimal training time at an early stage in their medical education. This result is in agreeance with results generated by other medical programs providing ultrasound training to students early on [2, 18]. The subjective data assessing self-confidence reported by individual participants (Fig. 1) and the scoring provided by the session instructor report similarly successful results. Students were able to obtain images of diagnostic quality in 97.7% (301 of 308) of the total ultrasound-related tasks they were asked to perform during training. The instructor reported that approximately half of the 7 unsuccessful attempts to acquire target views made by participants were directly the fault of the participant due to poor tactile technique (not pushing hard enough with the probe, not rotating/tilting the probe sufficiently to bring target object clearly into view) that was not isolated to any specific sonographic view across participants who struggled. The remaining half of the unsuccessful attempts were either due to the presence of excessive bowel gas or exceptionally difficult acoustic windows in the subjects being scanned.

Topics covered in training sessions (see Table 1) were selected specifically because they include target views that are commonly used throughout many clinical disciplines in point-of-care-ultrasound [3–5, 7, 12]. Our goal in making these selections was to focus on ultrasound skills that were likely to be useful in the very early stages of residency and perhaps even during clinical clerkships. Overall, participants reported in their responses to question 24 that our ultrasound training program was an enjoyable hands-on experience that deepened their enthusiasm for and understanding of medicine.

### Study limitations

We would like to acknowledge some potential limitations of this study such as selection bias. Students volunteered to participate in our ultrasound training program, hence there is arguably a selection bias for highly motivated students, who could master ultrasound skills more easily or who may be more likely to perceive the experience positively. Additionally, we have considered that an elective course in ultrasound should contain several other components (such as assessment and lecture). These additional components would also take time that we did not include in our calculations of time required of students and faculty. We also were unable to provide any retention data for this project to evaluate how our training program impacts student performance during clerkships. Lastly, we acknowledge that although university ultrasound equipment remained locked-up and unavailable to students outside of scheduled training sessions, we cannot guarantee that none of the students supplemented their training with additional educational materials during this study.

## Conclusions

We have concluded that an effective ultrasound training program can be included in medical school curricula at low cost and with only small student and faculty time allotments. Students quickly attained training through six short sessions, which were held after scheduled classes. Sessions were scheduled in a flexible manner according to the needs of the students and availability of the session instructor. Due to the brevity of each session and convenience of the scheduling system, small groups of participants were able to complete the whole training program in a short span of 4 weeks. This type of training can easily be worked into brief portions of the academic year (such as the academic quarter), over the summer, during short periods of time designated for elective courses, or over other shorter breaks between semesters.

With a single ultrasound machine, a single instructor teaching ultrasound for an average of 8 h per week (faculty teaching time varied from 6 to 10 h per week with an average of 8 h per week due to the flexible extra-curricular scheduling methodology used) was able to train all 28 students enrolled in the study over a 4-week period. There was no need for numerous expensive machines or multiple instructors to deliver broad introductory coverage of many types of ultrasound scans. This type of brief but broad training program minimizes both cost and number of faculty required when compared to more robust programs that fully integrate ultrasound into undergraduate medical curricula. Our condensed training program may provide a practical method for integrating a short but comprehensive introductory ultrasound program into schools previously thought to be lacking the time and/or resources needed to offer ultrasound training to first-year medical students.

### Future directions

Since most medical schools have their students on campus for two years prior to clinical clerkships, we also plan to design and test additional types of condensed training programs for second-year medical students. These programs would focus on the evidenced-based methods in which ultrasound can be used to identify pathology as well as honing more specific and technical ultrasound skills. It is our intent to encourage skeptical medical education programs to include ultrasound training during both pre-clerkship years. We also intend to explore the findings of this study further by setting up a skill retention study. This would compare the performance of our study participants to the performance of a control group in ultrasound related tasks during clinical clerkships. It would also be useful to carry out similar measurement once these students have reached residency to evaluate retention and performance.
